# Alien Hand Syndrome Secondary to Acute Anterior Corpus Callosum Infarction Despite Dual Antiplatelet Therapy: A Case Report

**DOI:** 10.7759/cureus.101688

**Published:** 2026-01-16

**Authors:** Ibrahim Kaleel, Abed Kaleel

**Affiliations:** 1 Neurology, HeadworX Clinic, Waterloo, CAN; 2 Neurology, McMaster University, Waterloo, CAN

**Keywords:** acute confusion, emergency service, motor skills, neurology and critical care, stroke

## Abstract

Alien hand syndrome is an uncommon yet recognized complication of ischemic stroke. It is characterized by involuntary yet seemingly purposeful limb movements that occur without the patient’s volitional control. The syndrome is most commonly associated with injury to the corpus callosum or adjacent medial frontal regions, where interhemispheric motor integration takes place. We report the case of a 50-year-old woman with multiple vascular risk factors who developed an acute infarction of the right corpus callosum. She presented with difficulty initiating movement, confusion, transient visual hallucinations, and involuntary movements of the left upper extremity that interfered with voluntary right-hand tasks, consistent with intermanual conflict. CT head and MRI of the brain confirmed ischemic stroke involving the body, genu, and splenium of the right corpus callosum. She was managed with supportive care with continuation of dual antiplatelet therapy, and subsequent inpatient rehabilitation. This case highlights the importance of recognizing alien hand syndrome as a potential complication of callosal stroke in patients with atypical post-stroke motor behavior.

## Introduction

Alien hand syndrome is a rare neurological disorder affecting voluntary motor control, in which patients experience involuntary movements of a limb that oppose or interfere with intended actions [1,2]. Lesions involving the corpus callosum or bilateral medial frontal cortices disrupt interhemispheric coordination [2,3]. The callosal variant commonly manifests as intermanual conflict, where one hand opposes or counteracts the actions of the other [2,4]. First described in patients with lesions involving the body and splenium of the corpus callosum, the syndrome has since been associated with vascular, neoplastic, degenerative, and traumatic etiologies as well [5]. Although ischemic stroke is a recognized cause, alien hand syndrome is often underrecognized in the acute setting and is instead often misattributed to delirium, seizures, or primary movement disorders [3,6]. We present a case of acute right corpus callosum infarction presenting with classic features of callosal alien hand syndrome.

## Case presentation

A 50-year-old woman with poorly controlled type 2 diabetes mellitus, hypertension, anemia, and coronary artery disease status post coronary artery bypass grafting presented with several days of difficulty initiating movements such as standing, transferring, and walking, despite preserved comprehension. Her family observed abnormal, non-rhythmic movements of the left upper extremity that appeared goal-directed but were not under voluntary control, a characteristic feature of alien hand syndrome [1,4]. They also reported episodes in which the patient’s left hand interfered with right-hand tasks during dressing and essentially undoing completed actions, consistent with intermanual conflict. 

On examination, the patient was alert and oriented with intact comprehension and fluent speech. Cranial nerve examination was normal. Coordination was intact without dysmetria. The patient endorsed a subjective sense of estrangement from the left hand, describing loss of ownership and involuntary actions perceived as independent of her intent, though motor strength was preserved throughout except for mildly reduced grip strength in the left hand. Muscle tone was normal, without tremor or spasticity. Sensory examination was intact bilaterally, and reflexes were symmetric. The left upper extremity exhibited intermittent involuntary movements that interfered with voluntary tasks, despite understanding commands.

Diagnostic assessment

**Figure 1:**
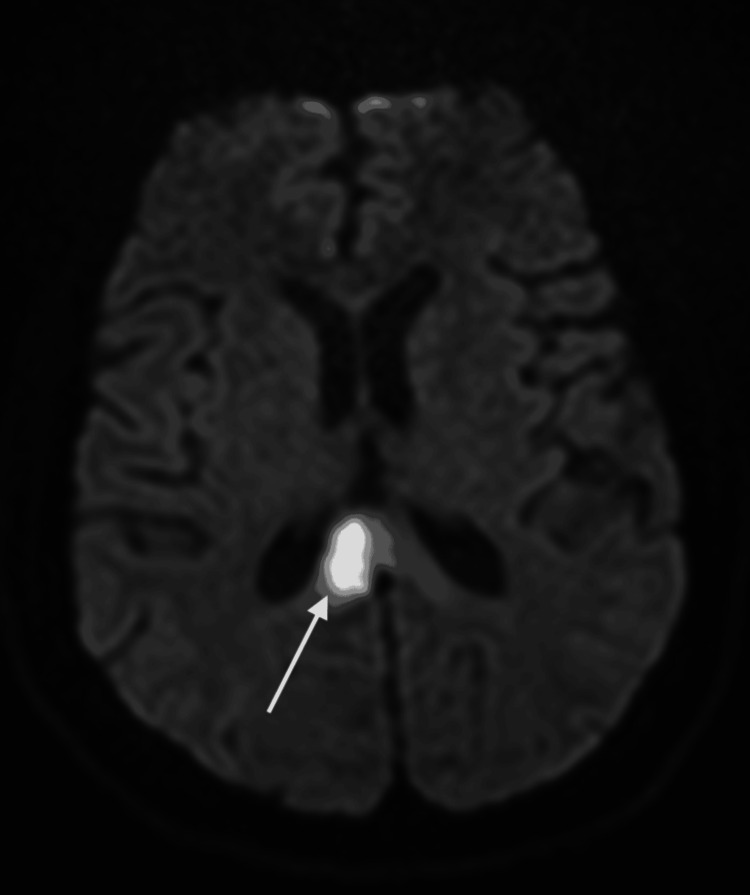
Restricted diffusion involving the splenium of the right corpus callosum, consistent with acute ischemic infarction

**Figure 2:**
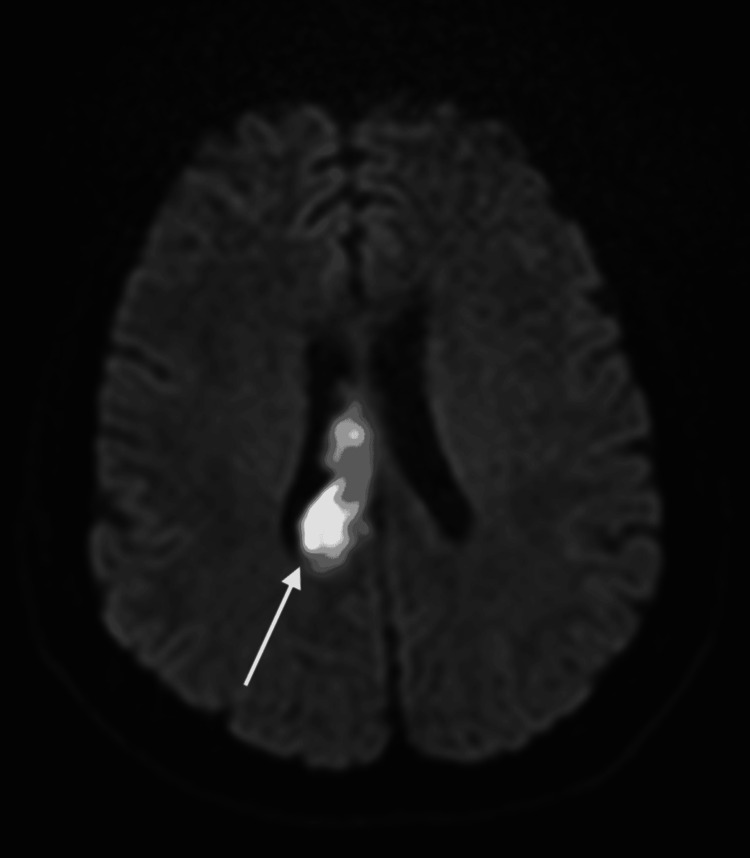
Restricted diffusion centered in the right corpus callosum extending from the body through the splenium, in keeping with an acute infarct

**Figure 3:**
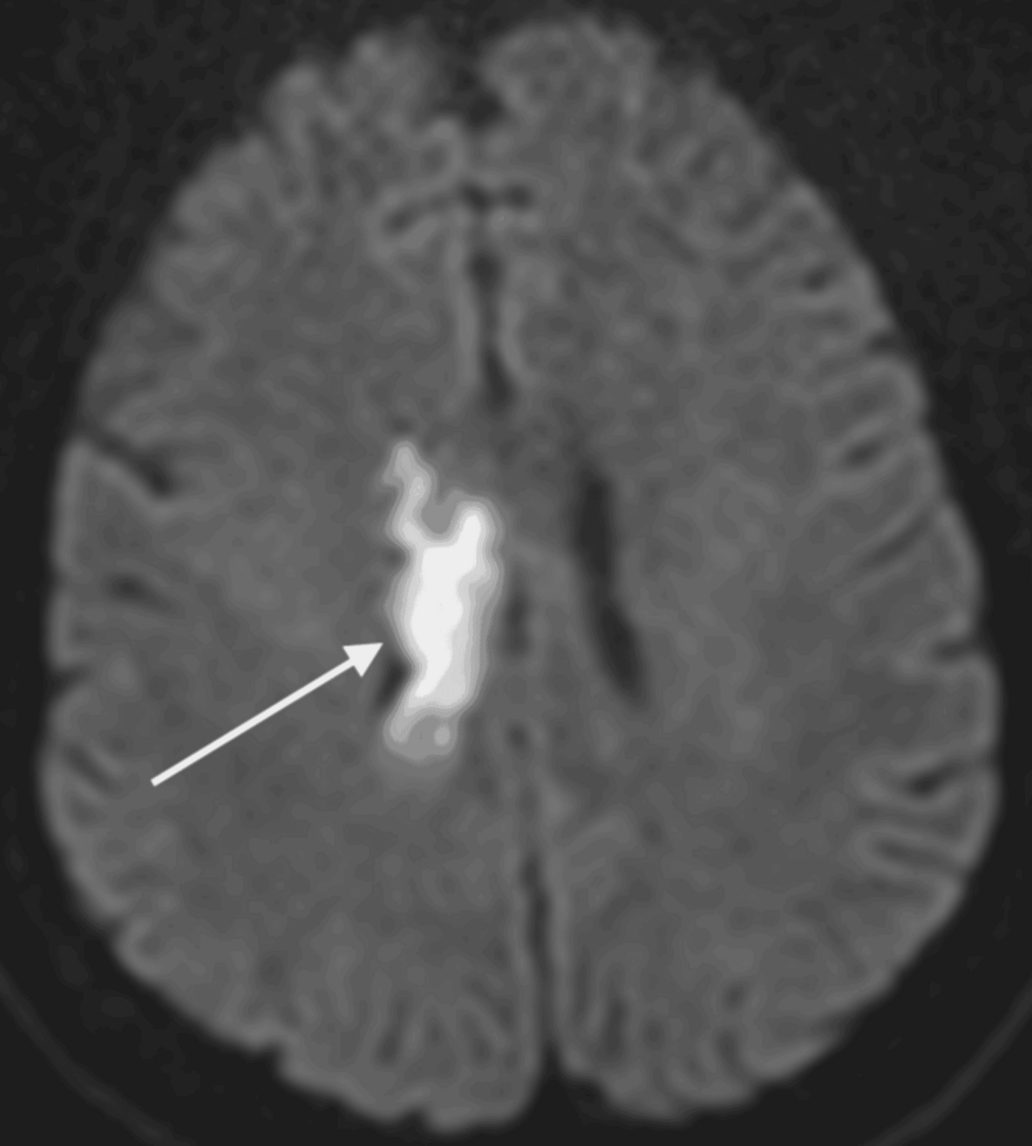
Abnormal diffusion restriction within the right corpus callosum, involving the splenium and genu, consistent with an acute territorial infarct

Non-contrast computed tomography (CT) of the head demonstrated infarction involving the body and splenium of the corpus callosum, with edema extending into the genu of the right corpus callosum. Magnetic resonance imaging (MRI) confirmed restricted diffusion consistent with acute ischemia (Figures 1-3), supporting the diagnosis of corpus callosum infarction [7,8]. CT angiography of the head and neck showed no significant carotid stenosis or other distal vascular compromise. Transthoracic echocardiography revealed normal left ventricular function without significant valvular disease. Based on clinical findings and concordant imaging, a diagnosis of callosal alien hand syndrome secondary to acute ischemic stroke was made.

Therapeutic intervention

The patient was not a candidate for thrombolytic therapy or endovascular intervention due to delayed presentation. She was continued on dual antiplatelet therapy in accordance with secondary stroke prevention guidelines for selected patients with recent ischemic events, previously initiated for secondary prevention of atherosclerotic cardiovascular disease, given her history of coronary artery disease status post coronary artery bypass grafting [9]. Management focused on supportive inpatient care and early initiation of structured physical and occupational rehabilitation [6,10]. Risk factor modification was emphasized, particularly glycemic control.

Follow-up and outcomes

During hospitalization, mild left-hand grip weakness persisted, but over time, no recurrent or severe episodes of intermanual conflict were observed prior to discharge. She was discharged thereafter to inpatient stroke rehabilitation for continued therapy and functional recovery [6,10].

## Discussion

Alien hand syndrome results from disruption of interhemispheric motor integration and is most commonly seen due to lesions of the corpus callosum or adjacent medial frontal regions, which are responsible for coordinating bilateral motor activity [1-3,5,11,12]. The callosal variant is characterized by intermanual conflict, in which involuntary movements of one hand interfere with the voluntary and intentional actions of the contralateral hand, a feature clearly demonstrated in our patient [2,4,11]. Prior case reports have also shown that ischemic lesions involving the body, genu, or splenium of the corpus callosum can produce this presentation, supporting the anatomical-clinical correlation observed on neuroimaging in our case [7,8].

Although ischemic stroke is a recognized cause of alien hand syndrome, it remains an uncommon presentation in the emergency setting and is therefore often underrecognized in the acute setting [3,6]. As patients may retain normal strength, sensation, and language while exhibiting impaired voluntary motor control, this often leads to misdiagnosis as apraxia, delirium, seizures, or primary movement disorders [3,6,13]. MRI is essential, as corpus callosum infarctions may be subtle or overlooked on initial computed tomography without magnetic resonance imaging confirmation [7,8].

To date, there is no established pharmacologic treatment for alien hand syndrome, and management is largely supportive, focusing on treatment of the underlying cerebrovascular event and rehabilitation [1,6]. Our patient demonstrated gradual improvement in alien hand behaviors and functional interference during hospitalization, reinforcing the importance of early recognition and rehabilitation in optimizing recovery [6,10].

## Conclusions

Alien hand syndrome is a rare, but clinically important manifestation of corpus callosum stroke that may present with involuntary yet goal-directed limb movements and intermanual conflict in the setting of preserved strength and comprehension. In this case highlighting the callosal variant of alient hand syndrome, symptoms improved over the course of hospitalization with supportive care and rehabilitation, with resolution of severe intermanual conflict prior to discharge. Early recognition of this phenomenon may prevent misdiagnosis and allow timely initiation of rehabilitation strategies that may improve functional outcomes and quality of life.
